# Machine Learning for Differential Diagnosis Between Clinical Conditions With Social Difficulty: Autism Spectrum Disorder, Early Psychosis, and Social Anxiety Disorder

**DOI:** 10.3389/fpsyt.2020.00545

**Published:** 2020-06-19

**Authors:** Eleni A. Demetriou, Shin H. Park, Nicholas Ho, Karen L. Pepper, Yun J. C. Song, Sharon L. Naismith, Emma E. Thomas, Ian B. Hickie, Adam J. Guastella

**Affiliations:** ^1^Autism Clinic for Translational Research, Child Neurodevelopment and Mental Health Team, Brain and Mind Centre, Children’s Hospital Westmead l Clinical School, Faculty of Medicine and Health, University of Sydney, Sydney, NSW, Autstralia; ^2^School of Psychology, University of Sydney, Sydney, NSW, Autstralia; ^3^Youth Mental Health Unit, Brain and Mind Centre, Central Clinical School, Faculty of Medicine and Health, University of Sydney, Sydney, NSW, Autstralia

**Keywords:** autism spectrum disorder, cognition, differential diagnosis, early psychosis, machine learning, social anxiety disorder

## Abstract

Differential diagnosis in adult cohorts with social difficulty is confounded by comorbid mental health conditions, common etiologies, and shared phenotypes. Identifying shared and discriminating profiles can facilitate intervention and remediation strategies. The objective of the study was to identify salient features of a composite test battery of cognitive and mood measures using a machine learning paradigm in clinical cohorts with social interaction difficulties. We recruited clinical participants who met standardized diagnostic criteria for autism spectrum disorder (ASD: n = 62), early psychosis (EP: n = 48), or social anxiety disorder (SAD: N = 83) and compared them with a neurotypical comparison group (TYP: N = 43). Using five machine-learning algorithms and repeated cross-validation, we trained and tested classification models using measures of cognitive and executive function, lower- and higher-order social cognition and mood severity. Performance metrics were the area under the curve (AUC) and Brier Scores. Sixteen features successfully differentiated between the groups. The control versus social impairment cohorts (ASD, EP, SAD) were differentiated by social cognition, visuospatial memory and mood measures. Importantly, a distinct profile cluster drawn from social cognition, visual learning, executive function and mood, distinguished the neurodevelopmental cohort (EP and ASD) from the SAD group. The mean AUC range was between 0.891 and 0.916 for social impairment versus control cohorts and, 0.729 to 0.781 for SAD vs neurodevelopmental cohorts. This is the first study that compares an extensive battery of neuropsychological and self-report measures using a machine learning protocol in clinical and neurodevelopmental cohorts characterized by social impairment. Findings are relevant for diagnostic, intervention and remediation strategies for these groups.

## Introduction

Machine learning (ML) paradigms have facilitated the evaluation of complex datasets (1, 2) and provide a dynamic framework to enhance comparisons between groups that may share neurodevelopmental, clinical or cognitive profiles (3). In contrast to the traditional multiple regression methods, the ML algorithms are also capable of including many input variables with relatively smaller sample sizes (4, 5) and can handle both linear and non-linear interactions between variables. In medicine and psychology, the resultant algorithms have led to insights in clinical classification within (6) and between clinical cohorts (7), transdiagnostic subtyping of mental health symptoms (8) and comparative lifetime health outcomes (9). Such research may contribute to improved profiling of cohorts such as Schizophrenia (SCH) and Autism Spectrum Disorder (ASD) given shared genetic liability (10) and theorized common etiologies associated with social cognition (11) and executive function (EF) (12) processes.

The clinical sub-groups of SCH and ASD have drawn much debate about similarities and differences that might exist between the two diagnoses (13, 14). There is considerable empirical support of shared genetic, neurocognitive, and behavioral pathways between SCH and ASD (15–17). In both, co-morbidities appear higher than expected population outcomes (18) and impairments in cognitive function appear similarly in domains of social cognition (19) and EF (20). For ASD, diagnosis may be made as early as 18 months of age, however a proportion is diagnosed in adolescence/adulthood (21). The developmental course of psychosis is different, with a slow progression beginning with social withdrawal and early psychosis (EP) (22) that typically begins in later adolescence and early adulthood. In these cases, a third of people who develop EP will go on to develop SCH (23). There has been limited research exploring cognitive markers that may assist differential diagnosis. Such comparisons are particularly useful in early adulthood prior to the chronic manifestation of SCH symptoms to permit early differentiation of these disorders.

In this study we adopted the cognitive domains framework outlined in the Diagnostic and Statistical Manual of Mental Disorders (DSM-5) (24). The DSM-5 (24) defines six cognitive domains as key domains for the assessment of neurocognitive disorders. These are complex attention, EF, learning and memory, language, perceptual–motor function, and social cognition. Within this framework complex attention refers to the processes of sustained attention (capacity to maintain attention on a discrete task over prolonged period), divided attention (focusing on two tasks simultaneously), selective attention (focusing on a specific task and ignoring others) and information processing speed. While acknowledging the considerable debate on the conceptualization of executive function (EF) (25, 26), research presented in this paper adopts the fractionated view of EF as noted in the DSM-5. Specifically, EF is characterized by discrete domains representing higher order cognitive processes. The EF domains include mental/cognitive flexibility (ability to shift between concepts), inhibition (ability to inhibit a previously learned or prepotent response), planning (ability to execute a sequence of actions so that a desired goal is achieved) and working memory (ability to store and dynamically manipulate information in temporary STM) (27).

In studies of complex attention, impairment in sustained attention has been reported in ASD (28) and on a composite battery of attention measures in EP (29). A recent comparison between EP and ASD (20) showed the former was significantly more impaired on attentional processes. Complex or top/down attentional processes have been shown to be guided by frontal neural circuitry (30), impairment noted above may reflect atypical processing in the prefrontal cortex in the ASD and EP groups, respectively.

Empirical findings in cognitive domains—other than EF and social cognition—are mixed and, in part dependent on the modality studied (verbal versus visuospatial). Studies with participants diagnosed with ASD have reported impaired performance in verbal learning (31), visuospatial short term memory (STM) (28), whilst others, noted superior visual (32) and comparable verbal STM (31, 32). In populations with psychosis, verbal STM and learning have also been noted to be impaired (12, 33, 34) but there have been mixed results for visual learning (33, 34). A study examining language domain measures in ASD with a neurotypical comparison group (35) found no differences between them. For the perceptual-motor domain difficulties have been reported for EP (34) and ASD (36).

There is, a larger body of research examining social cognition and EF domains and their contribution to symptoms and disability in each of the ASD and EP cohorts. Lower- and higher-order social cognition (37) performance has been shown to be reduced in participants diagnosed with either EP or ASD. These include performance on tests of emotion recognition (38, 39) and theory of mind tasks (34, 40, 41). Reduced performance on neurocognition has been reported for EP (22, 42) and ASD (36, 43, 44). Specifically, in relation to EF, impairment in EP has been reported in attentional shifting (20) with mixed findings across other domains including working memory and abstract thinking (33, 45). A recent meta-analysis in ASD across six EF domains (44) points to broad executive problems, likely characterized by aberrant neural network connectivity (46).

While there is evidence of difficulties across some of these cognitive domains for EP/SCH and ASD, respectively, few studies have directly compared EP with ASD. There is however, a greater body of literature comparing ASD and SCH. The overall findings on shared and distinct pathways remain equivocal and are to some degree moderated by the type of assessment used (e.g. behavioral, imaging, physiological). Greater commonalities are observed when comparing ASD and SCH on behavioral measures of social cognition and EF. Using an extensive battery of social cognition and EF tasks (47) comparable impairment was reported between the two clinical groups that was significantly worse than the neurotypical control group. Similar findings were reported in a recent study (48) on a battery of social cognition tests across the domains of emotion recognition, social perception, mental state attribution, and attributional style. The comparison between a group with ASD and a mixed cohort with SCH or schizoaffective disorder revealed comparable levels of impairment. It was further noted that the few significant differences between groups were mediated by symptom severity. Findings of comparable performance on behavioral measures of social cognition tasks however are mixed. Highlighting the importance of using stimuli with greater ecological validity, comparable performance was observed between ASD and SCH on emotion recognition tasks when stimuli were presented within a realistic contextual background. Furthermore, both groups were impaired compared to the neurotypical control group (49). Despite these similarities it was noted that IQ was a significant moderator for SCH but not for the ASD group. Findings suggest different cognitive processes may mediate the observed outcomes. Further evidence of differences between the two conditions on a behavioral attribution style task were reported in a meta-analysis (50). Greater impairment was observed in the ASD cohort compared to SCH. It was further observed that the transition from first episode psychosis to SCH resulted in greater impairment in the use of mental states in the SCH group compared to the EP group.

More differences between ASD and SCH emerge when underlying neural mechanisms are investigated even when the two cohorts are comparable on behavioral task performance. Using a task of perspective taking, (51) comparable performance was reported between ASD and SCH on behavioral tasks. Imaging data however, revealed that the two groups were distinguished by different functional connectivity outcomes with greater local orbitofrontal connectivity in ASD compared to SCH. Similar discrepancy between behavioral and neural outcomes were reported in a study utilizing a social judgment task (52). Comparable performance on the behavioral measure was guided by distinct neural mechanisms in the amygdala and associated neural circuit clusters and differentiated between individuals with ASD and schizotypal personality disorder. In two studies Ciarramidaro and associates examined intention attribution (53) and facial affect recognition (54) comparing individuals with ASD and paranoid SCH. In the former study, different neural mechanisms were observed, verifying the hyper-hypo connectivity hypothesis (55), despite comparable difficulties in making attributions between the two groups. In contrast, the latter study did not identify significant neural or behavioral differences between the two groups on the task used (implicit negative affect recognition task).

These findings highlight that methodological design including diagnostic subtypes, transition stage of SCH (early or chronic psychosis), type of task and assessment mode, may contribute to the observed behavioral phenotype and underlying neural mechanisms. ML provides a methodology that allows for multiple variates to be assessed in a single design and can thus make a significant contribution to this field. Applying this methodology to a comparison between ASD and EP, prior to transition to psychosis and entrenchment of chronic symptomatology could provide significant insights on the neurodevelopmental basis of SCH and shared and distinct profiles between the two groups.

Furthermore, the ML methodology and focus on conditions with social impairment adopted in this study presents a novel approach for guiding research in this area. In particular, ML allows for the evaluation of multifactorial assessment outcomes, this is particularly important given potential biases in self/informant assessments (20, 56). Identifying discriminating behavioral profiles can provide a framework for investigating mechanisms underlying the reported shared and distinct phenotypes.

Few studies (19, 20) compared the EP and ASD groups with a clinical group that shares the social impairment phenotype but has generally intact cognitive and EF (57) such as Social Anxiety Disorder (SAD). The SAD group presents an important comparison cohort given that EP (58) and ASD (59) are both associated with substantially elevated levels of social anxiety reported at 25% and 50%, respectively. In addition, there is a period of prodromal features that are difficult to distinguish between SAD and early psychosis (60). A comparison between the three groups could facilitate discriminating profiles and aid diagnosis.

The broad goal of this study was to use ML on a large dataset of multiple cognitive domain measures and mood self-appraisals. The aims were to identify differentiating profiles between neurodevelopmental (EP, ASD), clinical (SAD) and neurotypical (TYP) comparison groups. Identifying discriminating profiles between these conditions would facilitate diagnosis and early intervention.

Our assessment battery included multiple measures across the domains of complex attention, executive function, learning and memory, perceptual-motor function, and social cognition. Self-report measures of depression, anxiety, and stress were also included in the study given research evidence demonstrating high levels of co-morbid depression in SAD (48%) (61) with reported range between 35% and 70% (62, 63). Comparable rates of depression comorbidities (54%) have been reported for EP (64). In adults with ASD, the rate of depression disorders range from 38% to 70%, while the rate of anxiety disorders ranges from 50% to 65% (65, 66). To our knowledge, this is the first study to compare EP and ASD with SAD and a non-clinical comparison group across broad cognitive domains and affective states.

The first aim was to identify a profile that may distinguish between the combined social impairment cohort and control group. The second aim was to identify variables that differentiated the neurodevelopmental cohort from the SAD group. The third aim was to determine whether each of the EP, ASD, and SAD groups could be distinguished on a subset of measures from the other clinical groups and from each other. We predicted that the neurotypical control group would be distinguished from the social impairment cohort on self-appraisal measures of depression and anxiety, given the reported high comorbidity rates in the clinical groups. Second, we predicted that the neurodevelopmental cohort would be distinguished from the clinical comparison group on measures of attention, psychomotor speed, social cognition, EF, and visuomotor performance. This is based on literature findings that these domains are generally intact in SAD (57) but impaired in ASD (28, 31) and EP (34). Third, we predicted that the EP and ASD groups would be distinguished from each other on measures of complex attention given empirical support for impaired neural circuitry underpinning attention networks in EP (67). In their review, Wood and associates showed that attentional switching predicted transition from EP to SCH. This may be a useful marker for differential diagnosis. No specific predictions were made for the comparisons of ASD versus SAD/EP and EP versus SAD/ASD.

## Methods

### Ethics

Ethics approval was given by the University of Sydney Ethics Committee (Protocol number 2013/352). Informed consent was obtained from each of the participants by postgraduate research students and trained clinicians.

### Participants

Our dataset consisted of clinical participants who have met standardized diagnostic criteria for ASD (N = 62), EP (N = 48) or SAD (N = 83). Participants were sequential referrals from the Autism Clinic for Translational Research, Anxiety Clinic, and headspace clinics, at the Brain and Mind Centre, University of Sydney. Neurotypical control study volunteers (TYP = 43), were recruited separately through advertising at university websites. Clinical diagnoses were based on standardized diagnostic instruments (ADOS (68), ADIS-IV/V (69), SCID-I (70), PANS (71), and IQ was assessed based on scores on the WASI (72) or WTAR (73). Participants were excluded if IQ was below 70, prospective TYP participants were excluded if they reported past or current mental health diagnosis, or of they scored above cut-offs on screening instruments of depression, anxiety/social anxiety, stress or autism, [DASS-21 (74), SIAS (75), AQ-10] (76). Details of the diagnostic and assessment batteries and associated cognitive domains are presented in **Table 1**.

**Table 1:** Summary of assessment measures.

Assessment Type	Domain	Assessment Test	Outcome Measures and Interpretation
**Clinical and screening measures**			
Semi-structured, standardized assessment of autistic symptoms	Social Interaction and Communication Restricted and Repetitive Behaviors	**ADOS-2 (**68**)** Autism Diagnostic Observation Schedule – 2nd edition	symptom severity
Self-report measure of autistic traits and capacity to identify and understand social cues and engage in social interaction	Social Awareness Social Cognition Social Communication Social Motivation Restricted Interests and Repetitive Behavior	**SRS-2 (**77**)** Social Responsiveness Scale	Outcome measures on overall score and on each of the clinical scales -Higher scores, more autistic traits
	Social Anxiety	**ADIS-IV (**69**)** Anxiety Disorders Interview Schedule for DSM-IV.	–
	Schizophrenia	**SCID-I (**70**)** The Structured Clinical Interview for DSM-IV Axis I Disorders (SCID-I)	–
	Schizophrenia	**PANS (**71**)** Positive and Negative Symptoms Scale	–
**Symptom severity measures (self-report)**			–
	Depression Anxiety Stress	**DASS-21 (**74**)** Depression Anxiety Stress Scale	Outcome measures on total score and on each of the scales -Higher scores, greater severity
	Emotional Reactivity Cognitive Empathy Social Skills	**EQ (**78**)** Cambridge Behavior Scale Abbreviated Empathy Quotient	Outcome measures on total score and on each of the scales -Higher scores reflect higher levels of social cognition.
**Performance and self-report measures of social cognition**			-
		**Faux Pas Recognition Task (**79**)**	Outcome measures are: -Faux Pas Hit Rate -Faux Pas False Alarm Rate -D-Prime, a ratio of hits to false alarms -Faux Pas Questions Total Correct -Faux Pas Control Questions Correct -No Faux Pas Questions Total Correct -No Faux Pas Control Questions Correct -Higher scores, better social cognition -False rate,
	Emotion Recognition	**FEEST (**80**)** Facial Expressions of Emotions: Stimuli and Tests	Outcome measures are: -Total score -Score on each of the six basic emotions (happiness, surprise, fear, sadness, disgust, anger) Higher scores reflect higher levels of emotion recognition
Presentation of two series of social scenes, the first series without facial expressions and the second series with facial expressions	Emotion Recognition	**Movie Stills task (**81**)**	-Higher scores, better social cognition
	Emotion Recognition	**False Belief Picture Sequencing Task (**82**)**	Outcome measures are: -False belief -Social script -Capture -Mechanical Higher scores, better social cognition
	Emotion Recognition	**RMET (**83**)** Reading the Mind in the Eyes Test	-Higher scores, better emotion recognition
Reading of 50 words and assessment of correct pronunciation, may be subject to regional language variations in pronunciation	Overall Cognitive Ability	**WTAR (**73**)** Wechsler Test of Adult Reading	–
	Overall Cognitive Ability	**WASI (**72**)** Wechsler Abbreviated Scale of Intelligence	–
**Neuropsychological and self-report measures of cognitive function and cognitive domain per DSM-5 (**24**) framework**			–
**Complex Attention** involves sustained attention, divided attention, selective attention, and information processing speed **Executive function** involves planning, decision making, working memory, responding to feedback, error correction, overriding habits, and mental flexibility **Learning and memory** involves immediate memory, recent memory (free recall, cued recall and recognition memory) and long term memory **Language** involves expressive 0language (naming, fluency, grammar, and syntax) and receptive language **Social cognition** involves recognition of emotions and behavioral regulation			–
**Executive function** (self-report measure)		**BRIEF (**84**)** Behavioral Rating Inventory of Executive Function	-Higher score indicates negative self-report of EF
**Executive function^1^** **Language^1^** ^1^ DSM-5 defines fluency as a component of language but Fluency is generally accepted as an EF domain and was assessed as part of the EF battery	*Phonemic fluency *Semantic fluency	**COWAT (**85**)** Controlled Oral Word Association Test	-Higher score, better performance
**Executive function**	Cognitive flexibility	**TMT-B (**86**)** Trail Making Test-B	Outcome measure is completion time in seconds -Higher score worse performance
**Complex attention**	Attentional switching	**IED (**87**)** **Intra-Extra Dimensional Shift Test**	-Stages completed, higher score better performance -errors, higher score worse performance
**Complex Attention**	Sustained attention	**RVP (**87**)** Rapid Visual Processing Test	-Score range 0–1, score of “1” indicates perfect detection of target
**Complex Attention**	Information processing speed	**TMT-A (**86**)** Trail Making Test-A	Outcome measure is completion time in seconds -Higher score, worse performance
**Learning and Memory**	Verbal learning and memory	**LM – WMS-III (**88**)** Logical Memory Test Wechsler Memory Scale 3rd edition	-Higher score better performance
**Learning and Memory**	Visuospatial learning and memory	**PAL (**87**)** Paired Associate Learning	-Total errors, higher score worse performance
**Learning and Memory**	Verbal learning and memory	**RAVLT (**89**)** Rey Auditory Verbal Learning Test	-Higher score better performance
**Learning and Memory**	Visuospatial learning and memory	**SSP (**87**)** Spatial Span Test	-Total correct - higher score better performance -Total errors – higher score worse performance

### Assessment Battery

In this study, we utilized both neuropsychological (objective) and self-report (subjective) measures of social cognition, cognitive, and executive function, as well as self-report measures of affective states (depression, anxiety, and stress).

### Data Selection

**Figure 1:**
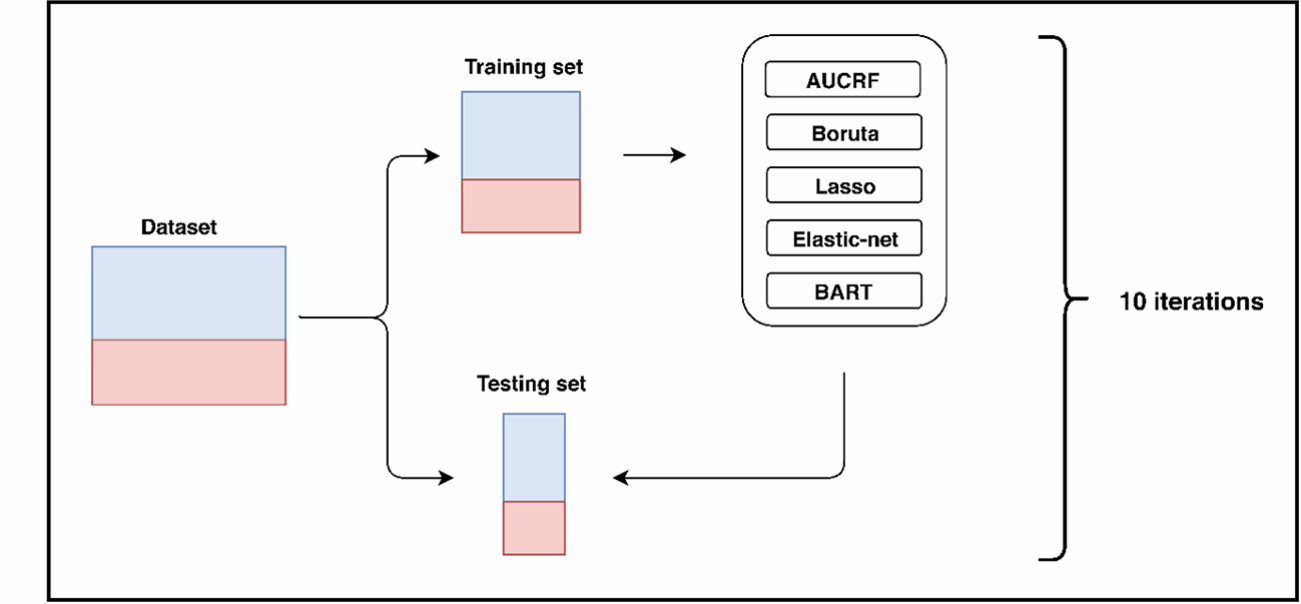
Data selection flowchart.

Patients and variables with more than 50% missing values were removed from the data set and the remaining missing data values were imputed with multivariate imputation with chained equations (MICE) (90) with 10 iterations using predictive mean matching for missing values. As shown in **Figure 1**, 10-fold cross-validation was applied to empirically assess the performance of the model built in imputed data sets. In each fold, 90% of the samples were used as the training set, and the remaining 10% were used for testing the generalizability of the models on unseen data. Ten-fold cross validation was used because it has been shown empirically to yield a reasonably low bias and modest variance (91, 92). Plausibility and consistency of the imputed values were visually inspected through density plots of the observed and imputed data, and the first imputed dataset was selected for downstream analysis.

### Machine Learning

We applied five different ML algorithms to build models that can classify between our groups of interest, because there is no best algorithm for all problems (93). A great model for one problem may not hold for another problem. In particular, we selected five algorithms that can also perform variable selection in order to ascertain a variable's contribution to the model: Area Under the Curve Random Forests (AUCRF) (94), Boruta (95), Lasso regression (96), Elastic net regression (97) and Bayesian Additive Regression Trees (BART) (98).

The performances of the ML models were assessed using the Area Under the Curve (AUC) and Brier Scores. The AUC represents the probability that a classifier will ranks a randomly chosen negative example lower than a randomly chosen positive example (99). The AUC is a widely used performance measure in machine learning, and is often used as the primary performance measure for binary classification (100). In the evaluation of ML algorithms, the AUC has been shown to be a statistically consistent and more informative metric as compared to other traditionally used metrics, such as accuracy, precision, and recall (101–103). AUC is known to be a more complete performance metric as compared to other traditionally used metrics. AUC values < 0.5 suggest no discrimination, 0.7 to 0.8 are considered acceptable, 0.8 to 0.9 are considered good, and ≥ 0.9 are considered outstanding (104). As the AUC only represents the ability of a prediction model to distinguish between classes (discrimination), the Brier score was additionally used to evaluate the magnitude of the error of the probability estimates (calibration and discrimination) for complementing the AUC (105). Brier scores range between 0 (perfect accuracy) and 1 (perfect inaccuracy). Higher AUC and lower Brier scores indicate which model is the most informative. For those with similar scores, repeatedly identified features would be the reliable and clinically informative discriminatory features. Furthermore, to provide additional insights and make the results comparable to other studies that report accuracy, precision (positive predictive value), and recall (sensitivity) as performance evaluation measures, we have included these three measures in our analysis (refer to **Table 3**).

### Machine Learning Algorithms

The AUCRF (94) and Boruta (106) algorithms are both based on the Random Forest (RF) (107) algorithm. RF uses bootstraps of samples to build a forest of decision trees with variables as nodes of the tree. Furthermore, RF has an internal variable importance ranking system that describes the decrease in node impurity. A higher-ranking variable is one that splits the samples into more pure groups.

AUCRF recursively builds RF models whilst eliminating the lowly ranked variables. The optimal set of variables is those used in the RF model with the best performance. For AUCRF, the metric for performance is the AUC which describes the model's true positive rate (sensitivity) and false positive rate (1-specificity) across different thresholds for binary classification.

Boruta uses RF to compare a variable's original importance score to its importance score from a permutation of that variable. Permutations break the relationship between the predictor and the response variables and, hence, are expected to decrease the predictive value of a variable. Variables with higher importance scores than in its permuted form are considered important. RF models were built with the optimal sets of variables as identified by AUCRF and Boruta, and tested to obtain performance metrics. For each RF model in AUCRF and Boruta and for each final RF model, we generated 1000 decision trees. The best variable for splitting at each level of each decision tree was identified from a random set of √p variables where p is the total number of variables. We also used internal 5-fold cross validation and a parameter tune length of 10 to identify the optimal value for λ (lambda). Lambda controls the strength of the penalization in Lasso and Elastic net and the balance between L1- and L2-regularization in Elastic net. Lasso (96) regression uses L1-regularization that penalizes coefficients with large absolute values in order to reduce overfitting. Lasso regression shrinks the coefficient of unimportant variables to zero and hence, effectively, performs variable selection. In contrast, Elastic net regression (97) employs a linear combination of L1-regularization and L2-regularization, which penalizes coefficients with large squared values. We used internal five-fold cross validation and a parameter tune length of 10 to identify the optimal value for λ which controls the strength of the penalization in Lasso and Elastic net, and α which controls the balance between L1- and L2-regularization in Elastic net.

In contrast to RF where trees are built from random bootstraps and independently, BART (98) employs a sum-of-trees approach. The Bayesian foundations of BART allows for the specification of regularization priors that ensures that each tree is weak and the use of Bayesian back-fitting (108) to fit trees iteratively. Variable selection with BART involves comparing the variable's inclusion proportions, which reflects the frequency of which the variable is chosen to be the split node, against a null distribution created from multiple permutations of the variable.

## Results

### Sample Description

Demographic characteristics are summarized in **Table 2**. In total, 236 participants were included in the study with mean age X = 22.7 years (SD 5.8), 96 (40.7%) were female. No significant differences were observed between the cohorts with regard to age, gender, and years of education (p > 0.05).

**Table 2:** Demographic descriptive statistics by diagnosis.

	All (n = 236)	Control (n = 43)	SAD (n = 83)	ASD (n = 62)	EP (n = 48)	Significance
**Age in years**	22.72 (5.83)	23.21 (5.84)	22.34 (6.15)	22.63 (5.55)	23.08 (5.76)	H(3) = 2.567, p = 0.463
**Gender female (%)**	96 (40.7%)	21 (48.8%)	28 (33.7%)	21 (33.9%)	26 (54.2%)	χ2(3) = 7.654, p = 0.054
**Education in years**	13.02 (2.19)	12.86 (2.00)	12.85 (2.30)	13.37 (2.45)	13.07 (1.81)	H(3) = 1.714, p = 0.634

H: Kruskal-Wallis Test for non-normal data.

### Model Performance

Classification performance for classifying between neurotypical controls and social impairment cohorts was good with mean AUCs greater than 0.87 (**Table 3**). All five algorithms performed similarly well with BART providing the highest mean AUC (0.92) and Boruta providing the lowest mean Brier Score (0.14).

**Table 3:** Classification performance on repeated cross-validation test sets.

	Control vs Clinical	SAD vs Neurodevelopmental (ASD and EP)	ASD vs SAD and EP	EP vs ASD and SAD	EP vs ASD	EP vs SAD	SAD vs ASD
**Test set AUC’s mean (SD)**							
**AUCRF**	0.891 (0.081)	0.752 (0.092)	0.676 (0.141)	0.776 (0.123)	0.747 (0.156)	0.825 (0.114)	0.741 (0.135)
**Boruta**	0.900 (0.076)	0.759 (0.090)	0.661 (0.137)	0.771 (0.124)	0.742 (0.163)	0.833 (0.117)	0.746 (0.133)
**Lasso**	0.871 (0.092)	0.729 (0.112)	0.718 (0.134)	0.712 (0.151)	0.724 (0.149)	0.746 (0.144)	0.780 (0.136)
**Elastic-net**	0.893 (0.076)	0.754 (0.099)	0.749 (0.118)	0.727 (0.143)	0.724 (0.141)	0.792 (0.139)	0.808 (0.125)
**BART**	0.916 (0.069)	0.781 (0.101)	0.735 (0.124)	0.777 (0.129)	0.759 (0.146)	0.827 (0.109)	0.782 (0.118)
**Test set Brier Scores mean (SD)**							
**AUCRF**	0.146 (0.042)	0.206 (0.037)	0.234 (0.046)	0.196 (0.042)	0.207 (0.056)	0.173 (0.044)	0.213 (0.051)
**Boruta**	0.138 (0.040)	0.204 (0.033)	0.237 (0.042)	0.197 (0.042)	0.206 (0.057)	0.170 (0.043)	0.209 (0.048)
**Lasso**	0.181 (0.058)	0.226 (0.047)	0.231 (0.058)	0.234 (0.046)	0.239 (0.061)	0.214 (0.068)	0.203 (0.052)
**Elastic-net**	0.153 (0.045)	0.208 (0.044)	0.224 (0.050)	0.234 (0.074)	0.237 (0.072)	0.194 (0.060)	0.185 (0.050)
**BART**	0.150 (0.026)	0.198 (0.032)	0.211 (0.028)	0.204 (0.028)	0.210 (0.032)	0.180 (0.034)	0.196 (0.034)
**Test set Accuracy’s mean (SD)**							
**AUCRF**	0.790 (0.096)	0.690 (0.094)	0.639 (0.109)	0.719 (0.103)	0.663 (0.142)	0.745 (0.115)	0.674 (0.115)
**Boruta**	0.801 (0.092)	0.685 (0.093)	0.625 (0.087)	0.707 (0.106)	0.668 (0.134)	0.747 (0.113)	0.693 (0.118)
**Lasso**	0.760 (0.094)	0.660 (0.115)	0.655 (0.110)	0.659 (0.114)	0.661 (0.130)	0.703 (0.121)	0.710 (0.130)
**Elastic-net**	0.784 (0.097)	0.689 (0.096)	0.684 (0.095)	0.681 (0.126)	0.654 (0.134)	0.735 (0.110)	0.732 (0.114)
**BART**	0.819 (0.079)	0.706 (0.095)	0.702 (0.103)	0.724 (0.102)	0.693 (0.143)	0.756 (0.109)	0.723 (0.105)
**Test set Precision’s mean (SD)**							
**AUCRF**	0.801 (0.210)	0.676 (0.129)	0.654 (0.144)	0.721 (0.126)	0.656 (0.221)	0.768 (0.197)	0.650 (0.183)
**Boruta**	0.810 (0.198)	0.682 (0.136)	0.635 (0.121)	0.707 (0.129)	0.643 (0.220)	0.757 (0.192)	0.681 (0.194)
**Lasso**	0.831 (0.209)	0.619 (0.151)	0.663 (0.147)	0.660 (0.139)	0.670 (0.220)	0.670 (0.196)	0.704 (0.193)
**Elastic-net**	0.817 (0.201)	0.644 (0.144)	0.706 (0.132)	0.683 (0.150)	0.666 (0.212)	0.705 (0.195)	0.707 (0.178)
**BART**	0.525 (0.142)	0.787 (0.107)	0.817 (0.093)	0.895 (0.075)	0.673 (0.192)	0.669 (0.169)	0.689 (0.150)
**Test set Recall’s mean (SD)**							
**AUCRF**	0.480 (0.145)	0.763 (0.107)	0.786 (0.099)	0.888 (0.076)	0.634 (0.195)	0.652 (0.162)	0.630 (0.158)
**Boruta**	0.501 (0.148)	0.756 (0.110)	0.782 (0.093)	0.884 (0.079)	0.645 (0.203)	0.655 (0.166)	0.657 (0.163)
**Lasso**	0.438 (0.134)	0.755 (0.132)	0.801 (0.099)	0.856 (0.088)	0.618 (0.168)	0.602 (0.165)	0.664 (0.171)
**Elastic-net**	0.472 (0.150)	0.786 (0.115)	0.810 (0.085)	0.866 (0.084)	0.619 (0.181)	0.659 (0.170)	0.701 (0.157)
**BART**	0.844 (0.188)	0.678 (0.139)	0.730 (0.117)	0.720 (0.125)	0.701 (0.207)	0.768 (0.183)	0.703 (0.178)

For classification between clinical and neurodevelopmental groups, the mean AUCs were lower than that between neurotypical controls and the combined social impairment cohort, which reflects the challenge in developing a classification tool between disorders. Mean AUCs for discrimination between the EP and ASD groups ranged from 0.72 to 0.76 with BART providing the highest mean AUC (0.76) and Boruta the lowest mean Brier Score (0.21).

### Variable Selection

**Figure 2:**
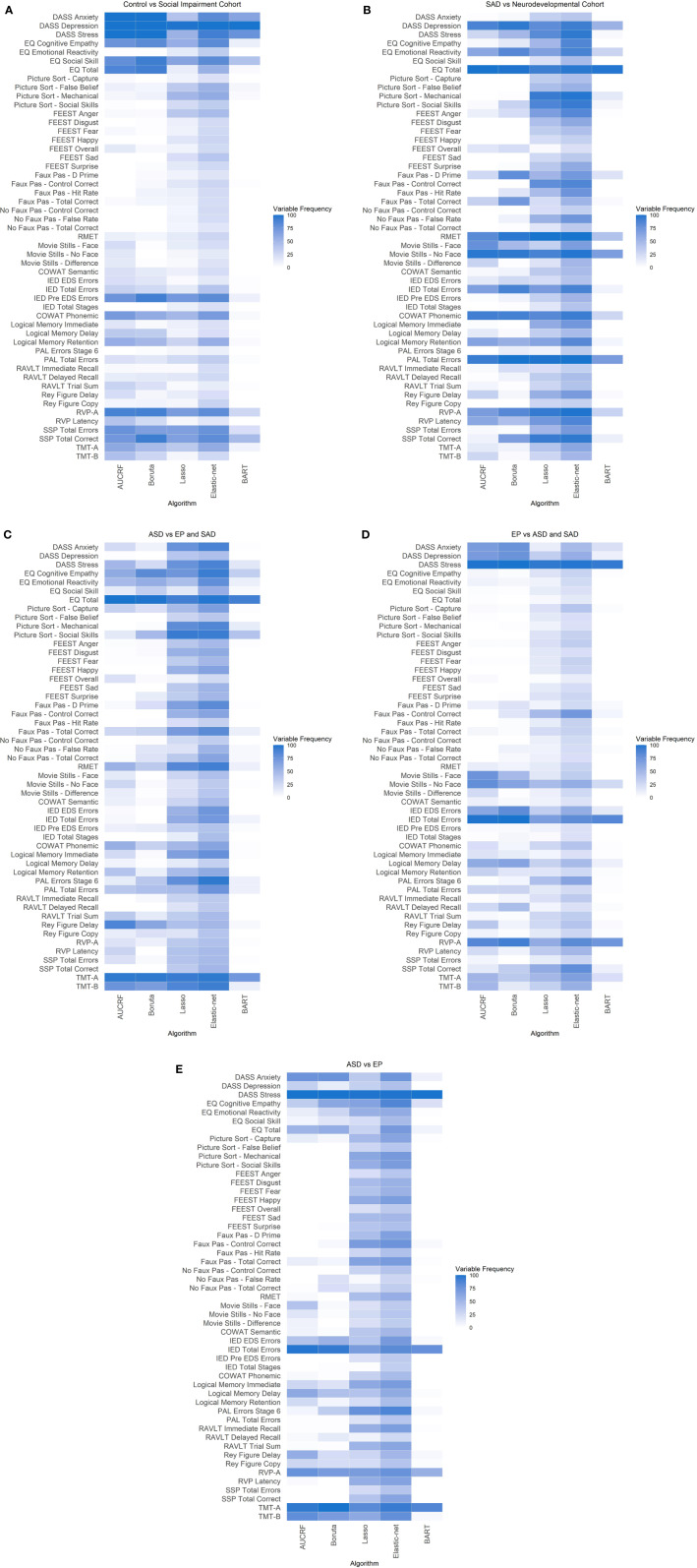
**(A)** Heatmap of Neurotypical Control vs Social Impairment Cohort. **(B)** Heatmap of SAD vs Neurodevelopmental Cohort. **(E)** Heatmap of ASD vs EP and SAD. **(D)** Heatmap of EP vs ASD and SAD. **(E)** Heatmap of ASD vs EP. **(A–E)** Variable frequency. Plot shows the frequency that each variable was selected for differentiating Control and Social impairment cohorts across the five algorithms with 10 times repeated 10-fold cross-validation. The darker color represents high frequency, while the lighter color represents low frequency.

**Figure 2A** illustrates the frequency that each variable was selected across the five algorithms with repeated cross-validation in the social impairment vs neurotypical control group. Self-report measures of depression, anxiety, and stress (DASS), social cognition measures of EQ-social skills, and the cognitive measure of visuospatial STM, best discriminated between the groups.

**Figure 3:**
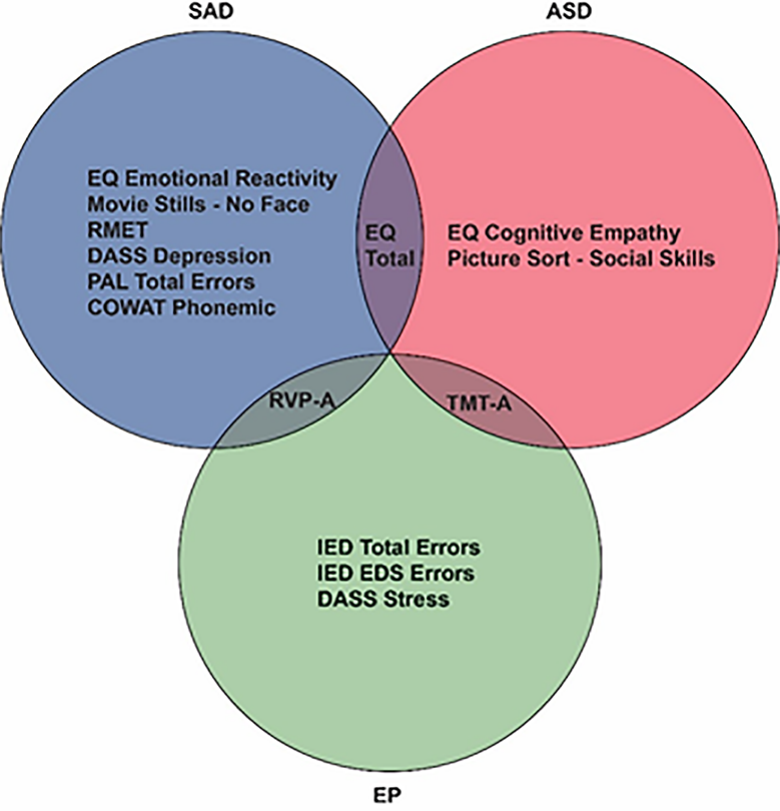
Venn diagram of distinguishing features.

**Figures 2B–E** and **Table 4** present the top variables identified from the variables input into the three models for differentiating diagnosis between the disorders of social impairments by all five algorithms. Discriminating variables were identified across cognitive domains and affective states. A summary of the key discriminating variables is presented in **Figure 3**.

**Table 4:** Variables discriminating between ASD, EP, SAD, and neurotypical controls.

Cohort	N	Variables
**TYP ∩ ASD/EP/SAD**	5	DASS Depression, DASS Anxiety, DASS Stress, EQ Social Skill, SSP, RVP-A
**SAD ∩ ASD/EP**	6	EQ emotional reactivity, Movie Stills—No Face, RMET, DASS Depression, PAL total errors, COWAT phonemic
**EP ∩ ASD/SAD**	3	IED Total Errors, IED EDS Errors, DASS Stress
**ASD ∩ EP/SAD**	2	EQ Cognitive Empathy, Picture Sort—Social Script
**ASD ∩ SAD**	1	EQ Total
**ASD ∩ EP**	1	TMT-A
**EP ∩ SAD**	1	RVP-A

Performance metrics like AUC and Brier scores represent how well the model differentiates between patients with different mental health problems. Based on the AUC and Brier scores, the most informative model can be identified. For example, the model for differentiating diagnosis between the SAD and neurodevelopmental group, the BART model showed the highest AUC = 0.781 and the lowest Brier score (0.198). However, other models also showed similar AUC and Brier scores. Features that are consistently selected by the best-performing model and these similarly performing models can be the recommendations for diagnosis and intervention strategies.

## Discussion

In this study, we used ML algorithms on a composite assessment battery to identify cognitive profiles that discriminate between clinical, neurodevelopmental, and neurotypical comparison groups. Our three hypotheses were that firstly, self-appraisal measures of depression and anxiety will differentiate the neurotypical group from the cohorts with social impairment. Second, the neurodevelopmental cohort will be distinguished from the SAD group on measures of attention, information processing, social cognition, EF, and visuomotor performance and third, the ASD and EP groups will be differentiated based on their performance on tasks of complex attention.

Our results showed that a reduced set of assessment measures differentiated between the comparison groups with good discriminative ability (AUC ≥ 0.7 and Brier score = 0.14–0.24). Our first hypothesis was confirmed in that depression, anxiety, and stress discriminated the combined social impairment cohort from the comparison control group. Two measures drawn from the social cognition and learning/memory domains (social skill and visuospatial short-term memory) complemented this profile. Our second hypothesis received partial support. Three of the predicted five cognitive domains (visual learning, social cognition, and EF), featured in the optimized profile discriminating between the neurodevelopmental groups and the SAD group. Depression was the other distinguishing feature. Finally, contrary to our third hypothesis, psychomotor speed rather than complex attention distinguished between the EP and ASD groups. Taken together, our research outcomes support and extend literature findings on distinguishing features of the SAD and neurodevelopmental (ASD/EP) groups. The results are particularly compelling given the high discriminative performance of the optimized profiles that emerged from an extensive battery across multiple cognitive domains and affective states.

The first finding of interest is that other cognitive domains in addition to EF and social cognition featured in the optimized profiles. The learning/memory domain measure of visuospatial memory contributed to the combined social impairment cohort versus control discriminating profile. This is surprising given that cognitive function in SAD (57) is generally intact, and thus not expected to differentiate this cohort from a neurotypical control group. The finding suggests that our combined social impairment cohort shares atypicalities in maintaining visual information in short term memory. There is evidence of reduced visual working memory capacity in EP (109) and ASD (110, 111) and our findings may in part reflect this. The shared profile with SAD however, points to more complex processes. A number of cognitive models predict that anxiety attenuates cognitive control and impairs working memory processes (112) including visual working memory (113). Our combined social impairment cohort is characterized by high levels of anxiety, and our findings may reflect the influence of anxiety on executive control.

Measures from learning, attention, and psychomotor speed domains featured in the optimized profiles that discriminated between clinical cohorts. Visual associative learning contributed to discriminating the neurodevelopmental from the SAD cohort. A closer examination of this profile indicated that although all groups were comparable on overall visual learning performance, the neurodevelopmental cohort made more errors. This may reflect impaired processes specific to EP and ASD including impaired visual working memory (109, 110) and slow processing speed (114, 115). Attentional processes were the most salient features that discriminated the EP group from the combined ASD/SAD cohort and EP from the SAD groups. Attentional neural circuitry in EP is clearly impaired in the course of illness (30) and indicates that it may have a unique role in early detection and differentiation. Psychomotor processing speed was the only distinguishing feature discriminating between EP and ASD groups. Research supports that processing speed is impaired in both groups (34, 114) however, different patterns of reaction time changes may apply. There is some evidence that processing speed in EP/SCH deteriorates in later age (116) whilst in ASD, processing speed has matured by adolescence (117) and is significantly impaired compared to neurotypical controls (114). The discriminating profile identified here may reflect different trajectory changes. The absence of measures from other cognitive domains in the EP/ASD comparison support that these two groups have a shared phenotype across most cognitive domains.

The second finding of interest is that phonemic fluency was the only EF measure that contributed to a profile discriminating between SAD and the neurodevelopmental cohort. Phonemic fluency performance is thought to be positively associated with intact frontal lobe function (118) and results may indicate frontal lobe alterations in EP (67) and ASD (119). Given that impairment in EF is noted for both EP (22) and ASD (36, 120) cohorts, greater prominence of EF measures would be expected. The limited role of our other EF measures in differentiating between the clinical groups suggests EF may have greater relevance as a transdiagnostic dimension of neurodevelopment (121).

Social cognition was a distinguishing feature for a number of optimized profiles. These measures featured in all profiles that included participants diagnosed with ASD, except for the ASD/EP direct comparison. Self-appraisals for social skill (a sub-scale of the EQ questionnaire that measures difficulty in social situations), differentiated the clinical cohort from the control group. Co-morbidity with SAD has been reported for each of the EP (122) and ASD (59) groups, and our finding of a shared profile feature likely reflects this. The neurodevelopmental cohort was distinguished from the SAD group on measures of basic emotion recognition (RMET task), identifying emotions in the absence of salient cues (movie stills task) and, in experiencing an appropriate emotion in response to another (self-appraisal of emotional reactivity/empathy). Finally, the ASD versus SAD profile distinguished between the two groups on the overall level of empathy (EQ questionnaire). These findings highlight the salience of social cognition in the neurodevelopmental cohort and particularly for the ASD group. Considered together with the limited prominence of EF features despite known EF deficits, it suggests that social cognition is a more important domain for discriminating the ASD group from other cohorts.

The prominence of mental health features (depression, anxiety, and stress) in the profile discriminating between the combined social impairment cohort and the control group, reflects the high levels of co-morbid depression (61, 64) and anxiety (122) reported for ASD, EP, and SAD. The inclusion of depression and stress self-appraisals in discriminating between the three clinical cohorts warrants further discussion and suggests that nuanced differences differentiate between the groups. The SAD group reported the highest levels of depression in our clinical cohort and EP the lowest levels of stress. Depression was the only affective state that discriminated the SAD versus neurodevelopmental cohort. This may reflect the high levels of co-morbid depression characterizing SAD (61). The lower levels of stress differentiating the EP group from ASD/SAD may reflect differences in symptom severity levels on presentation to our services. Acute positive psychotic symptoms in the EP cohort were controlled prior to inclusion in our services. The ASD and SAD participants however, would be experiencing a more acute profile of their respective symptoms. This may translate to the lower levels of distress reported by the EP group. Alternatively, lower stress in EP may reflect different levels of insight. There is research support that individuals with EP, (particularly those with more impaired cognitive function) have lower levels of insight (123) and may therefore report lower levels of stress.

### Limitations

There are a number of limitations in our study. First, although the sample size used in this study was larger than the suggested sample sizes of 75 to 100 for reasonable precision (4) the relatively small sample size of our cohort may reduce the parameters for trainability and cross-validations of our data. A larger sample size would be of benefit to further research. We also acknowledge the resources required to collect the detailed data we have. This is one of the largest studies with detailed information in the field to date. Second, our findings can only be attributed to individuals without intellectual disability, as we did not include any participants with an IQ below 70. Third, our findings include a number of features based on self-appraisals, and there is some question whether self-report appraisals by individuals with ASD are comparable to other cohorts (19, 20). Fourth, a number of participants in the diagnostic groups were being treated with medication, however, we were not able to control for medication use in this study. Fifth, we used 10-fold cross-validation to evaluate the classification performance of models and to identify the discriminating profiles between clinical, neurodevelopmental, and neurotypical comparison groups. Although this approach is considered as the most robust resampling technique to assess the accuracy and generalizability of models (124), the need for a more rigorous approach (external validation) has been emphasized to ensure the model generalizability (125). The present findings, therefore, need to be replicated in future studies with an independent large test set of completely unseen data in order to assess the generalizability of our ML models.

## Conclusions

The optimized profiles identified in our study highlight the importance of evaluating multiple cognitive domains when determining discriminating profiles between clinical groups. Further, they demonstrate that our combined social impairment cohort (ASD, EP, and SAD) is characterized by both shared and discriminating features. This has implications for diagnostic, intervention, and remediation strategies. The discriminating profiles can thus facilitate differential diagnosis particularly when clinical cohorts are characterized by comorbid mental health conditions and shared phenotypes. Conversely, the shared profile features, provide a framework for identifying transdiagnostic dimensions for intervention and remediation programs. The unique discriminating features (attention and empathy) that respectively characterized our EP and ASD cohorts potentially identify key target areas for early intervention programs. To-date there has been promising research on the effectiveness of intervention programs in improving social and non-social cognition in populations with ASD (126), EP (127) and SCH (128). In a study investigating cognitive support training in early psychosis (127) improvements were identified, however it was uncertain whether these were restorative or compensatory in nature. In cohorts with ASD, a recent study identified that higher levels of cognitive empathy mediated the positive influence of affective empathy on personal well-being (129). The researchers suggested that training programs on cognitive empathy could contribute to improvements in quality of life in ASD. Taken together these findings suggest that early intervention programs that target attention and empathy in the respective cohorts could contribute to improved functioning and potentially attenuation of symptoms.

To our knowledge, this is the first study that utilized measures across multiple cognitive domains and affective states. Our findings provide a framework for further research on shared and differentiating profiles of neurodevelopmental cohorts and cohorts characterized by social impairment.

## Data Availability Statement

The datasets generated for this study are available on request to the corresponding author.

## Ethics Statement

The studies involving human participants were reviewed and approved by The University of Sydney Ethics Committee. The patients/participants provided their written informed consent to participate in this study.

## Author Contributions

ED—conceptual design, literature review, data collection, manuscript preparation, manuscript revision. SP—conceptual design, data analysis, manuscript preparation, manuscript revision. NH—conceptual design, data analysis, manuscript preparation, manuscript revision. KP—data collection, manuscript revision. YS—manuscript revision. SN—manuscript revision. ET—data collection. IH—manuscript revision. AG—conceptual design, manuscript revision.

## Funding

This work was supported by the National Health and Medical Research Council (NHMRC) postgraduate scholarship to ED [GNT1056587], an NHMRC Australian Fellowship [APP 511921] to IH, and ARC Linkage Project grants [LP110100513; LP110200562], a National Health and Medical Research Council Career Development Fellowship [APP1061922], and a Project Grant [1043664; 1125449] to AG.

## Conflict of Interest

IH is a Commissioner in Australia's new National Mental Health Commission from 2012. He was a director of headspace: the national youth mental health foundation until January 2012. He was previously the chief executive officer (till 2003) and clinical adviser (till 2006) of beyondblue, an Australian National Depression Initiative. He is the Co-Director, Health and Policy at the Brain and Mind Centre that operates two early-intervention youth services under contract to headspace. He has led a range of community-based and pharmaceutical industry-supported depression awareness and education and training programs. He has led projects for health professionals and the community supported by governmental, community agency and pharmaceutical industry partners (Wyeth, Eli Lily, Servier, Pfizer, AstraZeneca) for the identification and management of depression and anxiety. He has received honoraria for presentations of his own work at educational seminars supported by a number of non-government organisations and the pharmaceutical industry (including Servier, Pfizer, AstraZeneca and Eli Lilly). He is a member of the Medical Advisory Panel for Medibank Private and also a Board Member of Psychosis Australia Trust. He leads an investigator-initiated study of the effects of agomelatine on circadian parameters (supported in part by Servier) and has participated in a multicentre clinical trial of the effects of agomelatine on sleep architecture in depression and a Servier-supported study of major depression and sleep disturbance in primary care settings.

The remaining authors declare that the research was conducted in the absence of any commercial or financial relationships that could be construed as a potential conflict of interest.
