# Supplementary material for: Machine Learning for Differential Diagnosis Between Clinical Conditions With Social Difficulty: Autism Spectrum Disorder, Early Psychosis, and Social Anxiety Disorder
Source: Front Psychiatry. 2020 Jun 19;11:545. doi: 10.3389/fpsyt.2020.00545 (PMC7319094; doi:10.3389/fpsyt.2020.00545)
Supplement: Supplementary file 1 [file Table_1.docx]

**Table S1: Group characteristics of cognitive and executive function, lower and higher order social cognition and mood severity measures.**

|  | 1. Control | 2. SAD | 3. ASD | 4. EP | Kruskal-Wallis H | Post hoc |
| --- | --- | --- | --- | --- | --- | --- |
| DASS Anxiety | 5.3 (5.18) | 16.39 (9.74) | 17.9 (10.88) | 11.25 (9.55) | F(3,236) = 52.108,  p < 0.001 | \| 4 > 1* \| \| --- \| \| 2 > 1*** \| \| 3 >1*** \| \| 2 > 4*  3 > 4** \| |
| DASS Depression | 5.4 (6.63) | 24.77 (11.23) | 22.26 (12.90) | 16.13 (12.70) | F(3,236) = 70.873,  p < 0.001 | \| 4 > 1*** \| \| --- \| \| 3 > 1*** \| \| 2 > 1*** \| \| 2 > 4** \| |
| DASS Stress | 6.94 (7.88) | 21.9 (9.74) | 23.26 (10.44) | 13.5 (10.73) | F(3,236) = 70.470,  p < 0.001 | \| 4 > 1* \| \| --- \| \| 2 > 1*** \| \| 3 > 1*** \| \| 2 > 4***  3 > 4*** \| |
| EQ Cognitive Empathy | 5.93 (2.72) | 4.31 (2.55) | 2.74 (2.33) | 4.21 (2.65) | F(3,236) = 35.163,  p < 0.001 | \| 4 > 3* \| \| --- \| \| 2 > 3** \| \| 1 > 3*** \| \| 1 > 4*  1 > 2* \| |
| EQ Emotional Reactivity | 5.33 (2.34) | 5.88 (2.26) | 4.52 (2.52) | 5.02 (2.18) | F(3,236) = 12.402,  p = 0.006 | \| 2 > 3** \| \| --- \| \|  \| \|  \| \|  \| |
| EQ Social Skill | 5.33 (3.34) | 2.77 (2.71) | 1.81 (2.09) | 2.93 (2.66) | F(3,236) = 33.785,  p < 0.001 | \| 1 > 3*** \| \| --- \| \| 1 > 2*** \| \| 1 > 4** \| |
| EQ Total | 16.58 (6.53) | 12.96 (4.55) | 9.08 (4.52) | 12.16 (5.60) | F(3,236) = 42.995,  p < 0.001 | \| 4 > 3* \| \| --- \| \| 2 > 3*** \| \| 1 > 3*** \| \| 1 > 4** \| |
| Picture Sort − Capture | 16.65 (4.91) | 17.05 (4.57) | 15.24 (4.80) | 16.71 (4.59) | F(3,236) = 5.249,  p = 0.154 |  |
| Picture Sort − False Belief | 19.74 (4.18) | 21.02 (3.50) | 19.65 (4.35) | 20.02 (4.00) | F(3,236) = 5.488,  p = 0.139 |  |
| Picture Sort − Mechanical | 21.37 (3.21) | 21.99 (3.13) | 22.42 (2.73) | 22.17 (3.53) | F(3,236) = 4.298,  p = 0.231 |  |
| Picture Sort − Social Skills | 23.05 (1.68) | 23.17 (1.70) | 21.95 (2.65) | 22.58 (2.46) | F(3,236) = 12.918,  p = 0.005 | \| 2 > 3** \| \| --- \| \|  \| \|  \| \|  \| |
| FEEST Anger | 7.6 (1.73) | 7.69 (1.72) | 6.73 (2.38) | 6.92 (1.87) | F(3,236) = 10.987,  p = 0.012 | \| 2 > 3* \| \| --- \| \|  \| \|  \| \|  \| |
| FEEST Disgust | 6.95 (2.3) | 6.81 (2.25) | 6.05 (2.83) | 6.17 (2.86) | F(3,236) = 3.812,  p = 0.283 |  |
| FEEST Fear | 6.28 (1.97) | 6.58 (2.19) | 6.03 (2.35) | 5.88 (2.31) | F(3,236) = 3.938,  p = 0.268 |  |
| FEEST Happy | 9.86 (0.41) | 9.82 (0.50) | 9.77 (0.49) | 9.69 (1.19) | F(3,236) = 1.262,  p = 0.738 |  |
| FEEST Sad | 7.88 (1.50) | 7.63 (1.84) | 7.16 (1.97) | 7.25 (2.19) | F(3,236) = 4.290,  p = 0.232 |  |
| FEEST Surprise | 9.14 (1.13) | 9.19 (1.08) | 8.55 (1.77) | 8.69 (1.67) | F(3,236) = 4.556,  p = 0.207 |  |
| FEEST Overall | 47.72 (4.05) | 47.71 (5.41) | 44.26 (6.99) | 44.58 (7.73) | F(3,236) = 14.048,  p = 0.003 | \| 2 > 3** \| \| --- \| \|  \| \|  \| \|  \| |
| Faux Pas − D Prime | 6.63 (2.51) | 6.68 (2.28) | 5.1 (3.26) | 5.88 (2.99) | F(3,236) = 12.363,  p = 0.006 | \| 2 > 3* \| \| --- \| \|  \| \|  \| \|  \| |
| Faux Ps − Control Correct | 4.91 (0.29) | 4.94 (0.24) | 4.85 (0.60) | 4.69 (0.78) | F(3,236) = 6.313,  p = 0.097 |  |
| Faux Pas − Hit Rate | 0.94 (0.10) | 0.94 (0.11) | 0.89 (0.21) | 0.88 (0.20) | F(3,236) = 4.313,  p = 0.230 |  |
| Faux Pas − Total Correct | 16.51 (2.85) | 16.37 (2.90) | 14.35 (4.61) | 15.13 (4.85) | F(3,236) = 7.815,  p = 0.050 |  |
| No Faux Pas − Control Correct | 4.93 (0.26) | 4.9 (0.30) | 4.79 (0.60) | 4.85 (0.41) | F(3,236) = 2.588,  p = 0.460 |  |
| No Faux Pas − False Rate | 0.09 (0.18) | 0.08 (0.14) | 0.2 (0.27) | 0.13 (0.25) | F(3,236) = 10.637,  p = 0.014 | \| 3 > 2* \| \| --- \| \|  \| \|  \| \|  \| |
| No Faux Pas − Total Correct | 9.07 (1.82) | 9.16 (1.44) | 7.97 (2.67) | 8.79 (2.29) | F(3,236) = 10.913,  p = 0.012 | \| 2 > 3* \| \| --- \| \|  \| \|  \| \|  \| |
| RMET | 26.51 (3.65) | 27.29 (4.03) | 23.23 (5.63) | 24.04 (5.71) | F(3,236) = 25.151,  p < 0.001 | \| 1 > 3* \| \| --- \| \| 2 > 3*** \| \| 2 > 4** \| |
| Movie Stills − Face | 11.41 (1.50) | 11.79 (1.71) | 11.05 (1.69) | 10.76 (1.76) | F(3,236) = 14.364,  p = 0.002 | \| 2 > 4*** \| \| --- \| \| 2 > 3* \| |
| Movie Stills − No Face | 10.18 (2.06) | 10.53 (1.81) | 9.49 (2.23) | 9.25 (1.73) | F(3,236) = 19.199,  p < 0.001 | \| 2 > 4*** \| \| --- \| \| 2 > 3* \| |
| Movie Stills − Difference | 1.22 (2.24) | 1.26 (1.71) | 1.56 (1.95) | 1.52 (1.68) | F(3,236) = 2.036,  p = 0.565 |  |
| COWAT Semantic | 21.74 (4.99) | 20.55 (4.81) | 19.06 (5.24) | 20 (4.05) | F(3,236) = 6.598,  p = 0.086 |  |
| IED EDS Errors | 6.47 (7.29) | 7.28 (9.15) | 9.19 (9.90) | 13.54 (10.85) | F(3,236) = 12.477,  p = 0.006 | \| 4 > 2** \| \| --- \| \|  \| \|  \| \|  \| |
| IED Total Errors | 15.49 (14.77) | 19.33 (18.53) | 27.05 (28.57) | 36.92 (30.37) | F(3,236) = 31.956,  p < 0.001 | \| 3 > 1* \| \| --- \| \| 4 > 1*** \| \| 4 > 2*** \| |
| IED Pre EDS Errors | 4.63 (2.33) | 6.39 (3.20) | 8.13 (5.74) | 7.69 (4.79) | F(3,236) = 21.505,  p < 0.001 | \| 2 > 1* \| \| --- \| \| 4 > 1*** \| \| 3 > 1*** \| |
| IED Total Stages | 8.74 (0.69) | 8.67 (0.73) | 8.44 (1.17) | 8.23 (1.29) | F(3,236) = 9.240,  p = 0.026 |  |
| COWAT Phonemic | 40.7 (9.44) | 37.36 (9.16) | 32.16 (11.56) | 32.77 (10.01) | F(3,236) = 24.412,  p < 0.001 | \| 2 > 3** \| \| --- \| \| 1 > 3*** \| \| 1 > 4** \| |
| Logical Memory Immediate | 41 (10.44) | 39.83 (12.65) | 38.39 (13.17) | 37.15 (9.86) | F(3,236) = 3.373,  p = 0.338 |  |
| Logical Memory Delay | 26.74 (7.21) | 25.18 (9.99) | 22.16 (10.58) | 21.48 (7.97) | F(3,236) = 12.196,  p = 0.007 | \| 1 > 4* \| \| --- \| \|  \| \|  \| \|  \| |
| Logical Memory Retention | 91.05 (12.99) | 85.3 (20.98) | 75.66 (24.47) | 79.53 (16.74) | F(3,236) = 17.170,  p < 0.001 | \| 1 > 3** \| \| --- \| \| 1 > 4** \| |
| PAL Errors Stage 6 | 1.56 (3.80) | 1.25 (1.89) | 6.4 (12.60) | 1.79 (3.00) | F(3,236) = 20.123,  p < 0.001 | \| 3 > 2* \| \| --- \| \|  \| |
| PAL Total Errors | 5.33 (7.44) | 4.76 (5.51) | 19.06 (32.40) | 12.31 (15.84) | F(3,236) = 10.053,  p = 0.018 | \| 3 > 2** \| \| --- \| \| 4 > 2** \| \| 3 > 1* \| \| 4 > 1* \| |
| RAVLT Immediate Recall | 12.63 (2.85) | 12.16 (2.83) | 10.98 (3.62) | 10.96 (3.17) | F(3,236) = 12.063,  p = 0.007 | \| 1 > 4* \| \| --- \| \| 1 > 3* \| |
| RAVLT Delayed Recall | 12.28 (3.51) | 11.75 (3.41) | 10.79 (3.76) | 10.69 (2.90) | F(3,236) = 12.378,  p = 0.006 | \| 1 > 4* \| \| --- \| \|  \| \|  \| \|  \| |
| RAVLT Trial Sum | 55.7 (10.76) | 55.04 (9.70) | 51.23 (12.17) | 53.06 (9.93) | F(3,236) = 5.652,  p = 0.130 |  |
| Rey Figure Delay | 21.67 (5.60) | 20.4 (6.21) | 18.2 (9.57) | 20.75 (6.60) | F(3,236) = 4.320,  p = 0.229 |  |
| Rey Figure Copy | 34.12 (2.11) | 33.24 (2.99) | 31.05 (6.24) | 32.48 (3.61) | F(3,236) = 9.080,  p = 0.028 | \| 1 > 4* \| \| --- \| \|  \| \|  \| \|  \| |
| RVP−A | 0.93 (0.04) | 0.91 (0.05) | 0.88 (0.07) | 0.87 (0.05) | F(3,236) = 37.009,  p < 0.001 | \| 2 > 4*** \| \| --- \| \| 1 > 4*** \| \| 2 > 3** \| \| 1 > 3*** \| |
| RVP Latency | 388.84 (51.47) | 388.36 (70.76) | 444.26 (138.27) | 450.35 (153.33) | F(3,236) = 6.460,  p = 0.091 |  |
| SSP Total Errors | 11.56 (5.64) | 14.99 (7.95) | 16.39 (7.22) | 16.13 (8.15) | F(3,236) = 14.993,  p = 0.002 | \| 2 > 1* \| \| --- \| \| 4 > 1* \| \| 3 > 1*** \| |
| SSP Total Correct | 7.6 (1.31) | 7.02 (1.26) | 6.37 (1.35) | 6.19 (1.36) | F(3,236) = 30.695,  p < 0.001 | \| 2 > 4** \| \| --- \| \| 1 > 4*** \| \| 2 > 3* \| \| 1 > 3*** \| |
| TMT−A | 22.38 (6.18) | 26.11 (9.36) | 35.45 (18.92) | 25.92 (8.81) | F(3,236) = 22.558,  p < 0.001 | \| 3 > 1*** \| \| --- \| \| 3 > 2** \| \| 3 > 4* \| |
| TMT−B | 52.12 (17.04) | 61.08 (22.59) | 81.73 (42.09) | 61.08 (23.3) | F(3,236) = 19.754,  p < 0.001 | \| 3 > 1*** \| \| --- \| \| 3 > 2* \| |
